# Genome Sequence of *Rickettsia tamurae*, a Recently Detected Human Pathogen in Japan

**DOI:** 10.1128/genomeA.00838-14

**Published:** 2014-09-04

**Authors:** Erwin Sentausa, Khalid El Karkouri, Caroline Michelle, Aurelia Caputo, Didier Raoult, Pierre-Edouard Fournier

**Affiliations:** Aix Marseille Université, URMITE, UM63, CNRS 7278, IRD 198, Institut National de la Sante et de la Recherche Medicale 1095, Marseille, France

## Abstract

*Rickettsia tamurae* is a member of the spotted fever group rickettsiae, which was reported in 2011 to cause human infections in Japan. We report the draft genome sequence of *R. tamurae* strain AT-1^T^, isolated from *Amblyomma testudinarium* ticks.

## GENOME ANNOUNCEMENT

Rickettsiae are obligate intracellular Gram-negative bacteria that cause rickettsioses, which are emerging infectious diseases. *Rickettsia tamurae*, a member of the spotted fever group rickettsiae, was first isolated from *Amblyomma testudinarium* ticks collected in Japan in 1993 (1). It was formally identified as a novel species by genetic and phylogenetic analyses in 2006 (2). In the same year, a spotted fever case from Laos was reported to be seroreactive for *R. tamurae* (3). However, it was not until 2011 that the first human infection case was confirmed using molecular and serological analyses in Japan (4). Here, we briefly describe the draft genome sequence from *R. tamurae* strain AT-1^T^.

The genomic DNA from *R. tamurae* AT-1^T^ (deposited in the Collection de Souches de l’Unite des Rickettsies [CSUR] under reference R1) was sequenced using an Illumina MiSeq platform (Illumina, San Diego, CA) with a mate-pair library. The CLC Genomics Workbench version 6.0.1 (CLC bio, Aarhus, Denmark) was used to perform quality trimming and *de novo* assembly of the reads. The resulting contigs were reordered using Mauve version 2.3.1 (5) and *Rickettsia montanensis* strain OSU 85-930 as a reference genome (GenBank accession no. CP003340.1). Potential coding sequences (CDSs) were predicted using AMIGene (6), and the assignment of protein functions was performed by searching against the RickBase (7), GenBank, and Pfam (8) databases using BLASTp (9), while ribosomal RNAs, tRNAs, and other RNAs were identified using BLASTn, tRNAscan-SE version 1.21 (10), and RNAmmer 1.2 (11), respectively. The orthologous genes between *R. tamurae* and *R. montanensis* were identified using OrthoMCL (12), with a BLASTp *E* value cutoff of 1 × 10^-5^ and a default Markov cluster (MCL) inflation parameter of 1.5.

The draft genome of *R. tamurae* AT-1^T^ consists of 27 contigs ranging in size from 266 to 319,774 bases, resulting in a total genome size of 1,453,216 nucleotides, with an average genome coverage of 140-fold and a G+C content of 32.5%. Two contigs (75,325 and 19,970 bp long) are putative plasmids with identity matches of 90% (32% coverage; *E* value, 0.0) to plasmid pMCE_1 from “*Candidatus* Rickettsia *amblyommii*” strain GAT-30V (accession no. CP003335.1) and 94% (44% coverage; *E* value, 0.0) to plasmid pRM from *R. monacensis* strain IrR/Munich (accession no. EF564599.1), respectively, when aligned using BLASTn. The chromosome contains 1,770 CDSs and, like other rickettsiae, 3 noncontiguous rRNAs (5S, 16S, and 23S rRNA), 33 tRNAs, and 3 other RNAs. The two plasmids contain 100 and 31 CDSs, respectively, but no RNAs.

The *R. tamurae* chromosome exhibits a high level of synteny to *R. montanensis*, with the exception of two inversions of 896 bp and 3,766 bp, respectively. Furthermore, several genes are lacking in the *R. tamurae* genome, including genes for a spore coat protein-like protein, isopentenyl-diphosphate delta-isomerase (*fni*), site-specific DNA methylase (*dam1*), a proline/betaine transporter (proP9_2), large extracellular alpha-helical protein, 3-hydroxyacyl-coenzyme A (CoA) dehydrogenase (*fadB*), competence protein F2 (*comF2*), PemK-like growth inhibitor, Sco_2_ protein precursor, and two toxin-antitoxin pairs.

### Nucleotide sequence accession numbers.

This whole-genome shotgun project has been deposited at DDBJ/EMBL/GenBank under accession numbers CCMG01000001 to CCMG01000027 (BioProject PRJEB6744).
